# Risk Factors of Thrombophilia-Related Mutations for Early and Late Pregnancy Loss

**DOI:** 10.3390/medicina60040521

**Published:** 2024-03-22

**Authors:** Ema Borsi, Ovidiu Potre, Ioana Ionita, Miruna Samfireag, Cristina Secosan, Cristina Potre

**Affiliations:** 1 Department of Internal Medicine, Discipline of Hematology, “Victor Babes” University of Medicine and Pharmacy, No. 2 Eftimie Murgu Square, 300041 Timisoara, Romania; borsi.ema@umft.ro (E.B.); ionita.ioana@umft.ro (I.I.) potre.cristina@umft.ro (C.P.); 2 Multidisciplinary Research Center for Malignant Hemopathies, “Victor Babes” University of Medicine and Pharmacy Timisoara, Eftimie Murgu Square 2, 300041 Timisoara, Romania; 3 Department of Internal Medicine, Discipline of Clinical Practical Skills, “Victor Babes” University of Medicine and Pharmacy, No. 2 Eftimie Murgu Square, 300041 Timisoara, Romania; samfireag.miruna@umft.ro; 4 Department of Obstetrics and Gynecology, Discipline of Obstetrics and Gynecology, “Victor Babes” University of Medicine and Pharmacy, No. 2 Eftimie Murgu Square, 300041 Timisoara, Romania; secosan.cristina@umft.ro

**Keywords:** thrombophilia, pregnancy loss, miscarriage, genetic testing

## Abstract

*Background and Objectives*: This retrospective cohort study investigates the role of genetic thrombophilia in pregnant women experiencing early pregnancy loss compared to those with late pregnancy loss. *Materials and Methods*: Participants were categorized into early and late pregnancy loss groups based on gestational age. A total of 156 patients were included, out of which 103 had early-trimester pregnancy losses and 96 had multiple miscarriages. *Results*: The study revealed a synergistic effect of Factor V Leiden (FVL G1691A) and Methylenetetrahydrofolate Reductase (MTHFR C677T) mutations (coefficient 3.42). Prothrombin (PT) G20210A and β-Fibrinogen 455 G>A mutations exhibited a significant interaction (coefficient 1.98). Additionally, MTHFR A1298C and Plasminogen Activator Inhibitor-1 (PAI-1 4G/5G) mutations showed a significant interaction (coefficient 1.65). FVL G1691A and Endothelial Protein C Receptor (EPCR) allele A1/A2 mutations also demonstrated a significant association (coefficient 2.10). Lastly, MTHFR C677T and Glycoprotein IIb/IIIa T1565C mutations interacted significantly (coefficient 1.77). Risk factor analysis identified several mutations associated with early pregnancy loss, including PAI-1 4G/5G homozygous (OR 3.01), FVL G1691A heterozygous (OR 1.85), and MTHFR A1298C heterozygous (OR 1.55). Both homozygous and heterozygous MTHFR C677T mutations were significant risk factors (OR 2.38; OR 2.06), as was PT G20210A homozygous mutation (OR 1.92). The PAI-1 4G/4G homozygous variant posed a risk (OR 1.36). Late pregnancy loss was associated with MTHFR A1298C homozygous mutation (OR 3.79), β-Fibrinogen 455 G>A heterozygous mutation (OR 2.20), and MTHFR A1298C heterozygous mutation (OR 2.65). Factor XIII G1002T heterozygous mutation (OR 1.18) and PAI-1 4G/5G homozygous mutation (OR 2.85) were also significant risk factors. EPCR allele A1/A2 (OR 1.60) and A2/A3 (OR 1.73) mutations were identified as significant risk factors for late pregnancy loss. Furthermore, FVL G1691A homozygous mutation, PT G20210A homozygous mutation, MTHFR C677T heterozygous mutation, MTHFR A1298C heterozygous mutation, and EPCR allele A1/A2 were identified as significant risk factors for multiple miscarriage. *Conclusions*: This study highlights significant interactions and risk factors related to genetic thrombophilia mutations in different types of pregnancy loss, contributing valuable insights for miscarriage management guidelines.

## 1. Introduction

Recurrent miscarriage, defined as two or more consecutive pregnancy losses before 20 weeks of gestation, affects approximately 1–5% of couples attempting to conceive [1]. The causes of recurrent miscarriage are multifaceted, including genetic, anatomical, hormonal, immunological, and environmental factors [2,3,4,5,6]. Among these, genetic factors, particularly thrombophilia, have garnered significant attention due to their potentially treatable nature and impact on pregnancy outcomes. Thrombophilia, both inherited and acquired, increases the risk of thrombosis and has been linked to adverse pregnancy outcomes, including recurrent miscarriage [7,8]. Nevertheless, the risk for thrombosis should be assessed in correlation with other triggering causes, such as malignancies, infectious diseases, and liver disease [9,10,11,12,13]. The identification of genetic factors for thrombophilia in pregnant women with a history of recurrent miscarriage could lead to targeted interventions, such as anticoagulation therapy, potentially improving pregnancy outcomes and reducing complications such as thrombosis, which can occur in up to 50% of the cases, depending on the study population [14,15,16].

Comprehensive genetic thrombophilia panel analysis encompasses testing for a range of genetic mutations associated with an increased risk of thrombosis, including Factor V Leiden, prothrombin gene mutation, MTHFR mutation, and deficiencies in protein C, protein S, and antithrombin [17,18,19]. Despite the known association between thrombophilia and adverse pregnancy outcomes, the role of comprehensive genetic thrombophilia screening in pregnant women with recurrent miscarriage remains underexplored, especially in the Romanian population. Some studies suggest that the identification and treatment of thrombophilia, among other correlated factors, can reduce the risk of recurrent miscarriage [20,21,22], while others argue against routine screening due to the low prevalence of detectable thrombophilia in this population and the unclear benefit of anticoagulation and other therapies [23,24,25].

The debate over the utility of comprehensive genetic thrombophilia panel analysis in this population underscores the need for prospective studies to better understand its impact on pregnancy outcomes considering the high costs of genetic testing. Such studies are essential to clarify the prevalence of thrombophilia in women with recurrent miscarriage and to determine whether targeted interventions based on types of mutations can improve outcomes. Furthermore, understanding the cost effectiveness and the psychological impact of thrombophilia screening and the associated assisted reproductive techniques is crucial for developing guidelines and recommendations for its use in clinical practice [26,27].

The hypothesis of this study is that comprehensive genetic thrombophilia panel analysis will identify a significant difference between pregnant women who experience a miscarriage in early pregnancy versus those affected by miscarriage in later pregnancy. The novelty of the study stands in the scarcely studied Romanian population of women affected by pregnancy loss in the context of a wide array of genetic tests that have not been carried out before in a large cohort of patients. By addressing these key questions, the study aims to contribute to the development of evidence-based guidelines for the management of miscarriage in the context of different thrombophilia mutations, potentially reducing the burden of this condition on affected women and their families.

## 2. Materials and Methods

### 2.1. Study Design and Settings

This retrospective cohort study aims to investigate the role of genetic thrombophilia in pregnant women who have first-trimester miscarriages compared with those who have second-trimester miscarriages. The study was conducted at the Clinical Municipal Hospital of Timisoara, which is affiliated with the Victor Babes University of Medicine and Pharmacy. This academic setting provided a comprehensive database of pregnant women diagnosed with miscarriage who underwent a comprehensive panel of genetic tests to assess thrombophilia gene mutations. The database search spanned between January 2018 and December 2023. All procedures were performed in accordance with the ethical standards of the institutional and/or national research committee and with the 1964 Helsinki Declaration and its later amendments or comparable ethical standards. The study received approval from the Institutional Review Board (approval no. 66/17 December 2023).

### 2.2. Participants

Participants were classified into two groups: “early pregnancy loss”, comprising pregnant women with a history of miscarriage before 13 weeks of gestation, and “later pregnancy loss”, including those who had a miscarriage in the second trimester (13 to 26 weeks of gestation). For the purposes of this study, the demarcation between early and late miscarriage was explicitly set at 13 weeks and 0 days (13 + 0), where losses occurring up to and including this gestational age were classified as early miscarriages, and those occurring afterward were considered late miscarriages [28]. Inclusion criteria for the study were (1) women aged 18 to 45 years; (2) documented history of miscarriage(s) within the study timeframe; (3) consent to participate in the study and for the use of their medical records for research purposes; (4) a documented thrombophilia genetic panel. Exclusion criteria included (1) known chromosomal or anatomical abnormalities of the fetus; (2) history of induced abortion; (3) presence of autoimmune diseases and antiphospholipid syndrome; (4) incomplete medical records; (5) lack of consent to provide access to personal and medical records; (6) presence of comorbid factors that are known to influence the coagulation balance, such as malignancies [29,30,31], recent surgical procedures [32,33,34,35], and different hematologic disturbances and medication regimens [36,37].

### 2.3. Variables and Definitions

The study focused on a comprehensive range of variables to establish precise diagnoses of thrombophilia among participants and to assess the status of pregnancy loss in line with the International Classification of Diseases (ICD-10) [38]. The collected variables included the following.

Maternal background data: age, body mass index (BMI), area of residence (urban or rural), relationship status, income level, education level, occupation, and comorbidities; Behavioral data such as smoking and alcohol consumption habits.

Obstetrical characteristics: gestational age at enrollment, total number of pregnancies, total number of term pregnancies, history of pregnancy loss detailed by gestational week, history of abortion (categorized as threatened, inevitable, complete, and missed), high obstetrical risk factors, pelvic infections, history of sexually transmitted diseases (STDs), infertility cases, and the use of assisted reproductive technologies (ART).

Laboratory parameters: thrombophilia screening performed, including tests for Factor V Leiden, prothrombin gene mutation, antithrombin deficiency, protein C and S deficiencies (total and free), plasminogen activator inhibitor-1 (PAI-1), Angiotensin-Converting Enzyme (ACE) deletion, deficiencies in Factors VII and XIII, β-fibrinogen polymorphism, glycoprotein Ia polymorphism, plasminogen and tissue-type plasminogen activator deficiencies, acquired activated protein C resistance, and MTHFR gene mutation.

Recurrent miscarriage was defined according to the American Society for Reproductive Medicine (ASRM) and the European Society of Human Reproduction and Embryology (ESHRE) guidelines as two or more consecutive pregnancy losses before the 20th week of gestation [2]. Pregnancy loss was defined as the spontaneous demise of a pregnancy before the fetus reaches viability, as per the World Health Organization (WHO) definition [39]. All patients received Folic Acid and Enoxaparin during the last pregnancy, with a dose of 0.40 UI/mL during all trimesters.

### 2.4. Laboratory Analysis

Blood samples were collected into vacuum tubes containing sodium citrate as an anticoagulant. These samples underwent centrifugation for ten minutes to produce platelet-depleted plasma, were immediately used for antithrombin activity analysis, and were stored at −80 °C for subsequent assays. The assay techniques included: (1) functional clotting assays used to assess activities of plasma proteins C and S; (2) Enzyme-Linked Immunosorbent Assay (ELISA) kits employed for measuring free protein S antigen levels; (3) Antithrombin activity determined using the Siemens diagnostic kit’s Berichrom^®^ (Siemens AG, München, Germany) [40] and lupus anticoagulant screening with lupus-sensitive activated partial thromboplastin time (APTT) and a dilute Russell’s viper venom time test [41]; (4) Cardiolipin and 2-Glycoprotein antibodies quantified through ELISA tests.

Diagnoses of protein C, S, or antithrombin deficiencies were based on activity or antigen levels falling below two-thirds (67%) of the threshold set for the 5th percentile, indicating severe deficiencies. DNA was extracted from peripheral blood leukocytes for allele-specific restriction enzyme testing to detect Factor V Leiden (FVL) and prothrombin G20210A mutations.

### 2.5. Statistical Analysis

Statistical analyses were conducted using IBM SPSS Statistics version 27.0 (SPSS Inc., Chicago, IL, USA). Data were initially screened for completeness and outliers, ensuring the integrity of the dataset for reliable analysis. Categorical variables, such as the presence of specific genetic markers, patient demographics (including area of residence, education level, and smoking/alcohol use), and obstetrical history (including history of pregnancy loss and ART use), were summarized using frequencies and percentages. Continuous variables, such as age, BMI, and gestational age at the time of loss, were described using means and standard deviations or medians and interquartile ranges, depending on the distribution of the data.

The normality of the distribution for continuous variables was assessed using the Shapiro–Wilk test. Variables adhering to a normal distribution were compared using the Student’s *t*-test for independent samples between cases and controls. For non-normally distributed variables, the Mann–Whitney U test was utilized. Categorical variables were analyzed using the Chi-square test or Fisher’s exact test, as appropriate, to compare the proportions between cases and controls.

To investigate the association between thrombophilia markers and late pregnancy loss, logistic regression analyses were performed. Univariate logistic regression was first conducted to identify potential risk factors associated with late pregnancy loss. A secondary analysis was performed by stratifying the number of miscarriages as one or multiple. Subsequently, multivariate logistic regression analysis was employed to adjust for potential confounders, including age, BMI, and other relevant factors identified in the univariate analysis. The selection of variables for the multivariate model was based on their statistical significance in the univariate analysis and their clinical relevance, as determined by the literature. The results are presented as odds ratios (OR) with 95% confidence intervals (CIs), with an alpha threshold of significance set at *p* < 0.05.

## 3. Results

The current study examined the demographics of 155 participants, encompassing 103 women who experienced early pregnancy loss and 52 women who faced late pregnancy loss. The analysis of body mass index (BMI) revealed an average BMI of 22.8 in the early pregnancy loss group and 23.6 in the late pregnancy loss group, with the difference not reaching statistical significance (*p* = 0.294).

Age distribution analysis indicated a higher percentage of younger women (20–30 years) in the early pregnancy loss group (35.14%) compared to the late pregnancy loss group (54.55%), and a greater proportion of women aged 31–40 years in the late loss group (61.36%) than in the early loss group (45.95%). However, these age-related differences did not achieve statistical significance (*p* = 0.081).

Residential patterns showed a higher proportion of urban dwellers among early pregnancy loss patients (79.6%) compared to late pregnancy loss patients (67.3%), and, conversely, a larger percentage of rural residents in the late loss group (32.7%) compared to the early loss group (20.4%). Despite these differences, the association between place of residence and pregnancy loss timing was not statistically significant (*p* = 0.092).

Educational background varied across the two groups, with the early loss group having a slightly higher percentage of college- or university-educated women (54.4%) compared to the late loss group (46.2%). However, differences in educational levels did not reach statistical significance (*p* = 0.163). The analysis of the number of miscarriages revealed no significant difference in the distribution between women with different numbers of losses in the early versus late pregnancy loss groups (*p* = 0.136).

Lastly, the presence of children in the household showed no significant difference between the two groups, with a majority of women in both groups not having children at the time of the study (89.3% in the early loss group and 90.4% in the late loss group, *p* = 0.837), as presented in Table 1.

The method of conception, i.e., naturally conceived, conceived through in vitro fertilization (IVF), or conceived through artificial insemination, showed no significant statistical difference between the early and late pregnancy loss groups (*p* = 0.354). Specifically, 79.6% of the early loss group and 75.0% of the late loss group had naturally conceived pregnancies, indicating that the mode of conception might not significantly impact the timing of pregnancy loss. Contraceptive use prior to pregnancy was prevalent in 60.2% of the early loss group and 61.5% of the late loss group, with the difference not reaching statistical significance (*p* = 0.871).

Regarding comorbid conditions, the analysis showed no significant differences in the prevalence of personal or family history of thrombosis, thyroid disease, and hypertension between the two groups. However, the incidence of diabetes was significantly higher in the early pregnancy loss group (15.5%) compared to the late pregnancy loss group (3.8%), with a *p*-value of 0.031, indicating a potential association between maternal diabetes and the risk of early pregnancy loss.

Laboratory analyses revealed significant differences in anti-factor X levels during the first trimester, with the early loss group exhibiting higher levels (0.33 ± 0.11 IU/mL) compared to the late loss group (0.29 ± 0.07 IU/mL), *p* = 0.018. This finding could suggest a differential role of anticoagulation in the pathophysiology of early versus late pregnancy loss. However, anti-factor X levels in the second and third trimesters and D-dimers levels across all trimesters did not significantly differ between the groups, as seen in Table 2.

The presence of the Factor V Leiden (FVL) G1691A heterozygous mutation was significantly higher in the early pregnancy loss group (36.9%) compared to the late pregnancy loss group (11.5%), with a *p*-value of less than 0.001. Similarly, the Prothrombin (PT) G20210A homozygous mutation was significantly more prevalent in the early loss group (26.2%) than in the late loss group (1.9%), with a *p*-value of less than 0.001.

The Methylenetetrahydrofolate Reductase (MTHFR) C677T homozygous mutation showed a significant discrepancy, being present in 12.6% of the early loss group and absent (0%) in the late loss group (*p* = 0.007). Moreover, the MTHFR C677T heterozygous mutation was significantly more common in the early loss group (65.1%) compared to the late loss group (28.8%), with a *p*-value of less than 0.001. The MTHFR A1298C heterozygous mutation also showed a significant difference, with a higher prevalence in the early loss group (60.2%) than in the late loss group (17.3%), with a *p*-value of less than 0.001.

The Plasminogen Activator Inhibitor-1 (PAI-1) 4G/5G homozygous mutation was found in 30.1% of the early loss group compared to 11.5% of the late loss group, with a *p*-value of 0.010. The PAI-1 4G/5G heterozygous mutation also showed a significant difference, being more prevalent in the early loss group (47.6%) compared to the late loss group (21.2%), with a *p*-value of 0.001. Additionally, the PAI-1 4G/4G homozygous mutation was significantly associated with early pregnancy loss (14.6% in the early loss group vs. 1.9% in the late loss group, *p* = 0.014), as described in Table 3.

The Factor V Leiden (FVL) G1691A homozygous mutation presented a notable difference between groups, with a significantly higher prevalence observed in women with multiple miscarriages (13.5%) compared to those with a single miscarriage (1.7%), yielding a *p*-value of 0.012. Similarly, the Prothrombin G20210A homozygous mutation was more prevalent in the multiple miscarriages group (16.7%) than in the single miscarriage group (20.3%), with a *p*-value of 0.016.

The MTHFR C677T heterozygous mutation also showed a statistically significant difference, being more prevalent in the multiple miscarriages group (60.4%) compared to the single miscarriage group (40.7%), with a *p*-value of 0.017. Additionally, the MTHFR A1298C heterozygous mutation was significantly more common among women with multiple miscarriages (52.1%) than those with a single miscarriage (35.6%), with a *p*-value of 0.045.

Another important finding was the association of the Endothelial Protein C Receptor (EPCR) allele A1/A2 with multiple miscarriages, where it was significantly more prevalent (49.0%) compared to in the single miscarriage group (25.4%), with a *p*-value of 0.003, as presented in Table 4.

One of the most notable findings was the interaction between the homozygous mutations of Factor V Leiden (FVL G1691A) and MTHFR C677T, which exhibited a coefficient of 3.42 with a *p*-value of 0.003. This significant interaction suggests a synergistic effect between these two genetic mutations that increases the risk of multiple miscarriages, indicating that individuals with both mutations may be at a substantially higher risk.

Additionally, the interaction between the Prothrombin (PT) G20210A homozygous mutation and the β-Fibrinogen 455 G>A heterozygous mutation was associated with a coefficient of 1.98 and a *p*-value of 0.009. The heterozygous mutations of MTHFR A1298C and Plasminogen Activator Inhibitor-1 (PAI-1 4G/5G) also demonstrated a statistically significant interaction, with a coefficient of 1.65 and a *p*-value of 0.015.

An interaction between the heterozygous mutation of FVL G1691A and the Endothelial Protein C Receptor (EPCR) allele A1/A2 was observed, presenting a coefficient of 2.10 and a *p*-value of 0.006. Furthermore, the interaction between the homozygous mutation of MTHFR C677T and the heterozygous mutation of Glycoprotein Iib/IIIa T1565C showed a coefficient of 1.77 and a *p*-value of 0.021, as presented in Table 5.

The analysis revealed that the homozygous variant of Plasminogen Activator Inhibitor-1 (PAI-1 4G/5G) significantly increased the risk of early pregnancy loss, with an odds ratio (OR) of 3.01 (*p*-value < 0.001). Similarly, the Factor V Leiden G1691A heterozygous mutation was identified as a significant risk factor, with an OR of 1.85 (*p*-value = 0.002). The MTHFR A1298C heterozygous mutation also emerged as a significant risk factor, with an OR of 1.55, (*p*-value = 0.028).

**Figure 1:**
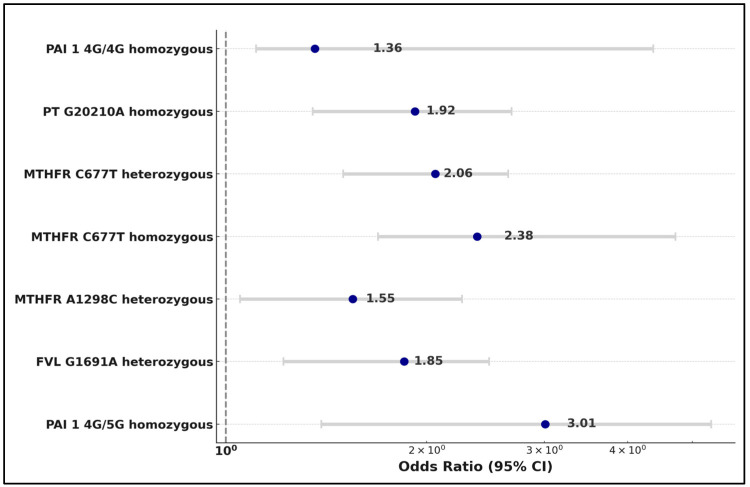
Risk factor analysis for early pregnancy loss.

Moreover, both the homozygous and heterozygous variants of the MTHFR C677T mutation were significantly associated with early pregnancy loss. The homozygous variant showed an OR of 2.38 (*p* < 0.001), while the heterozygous variant exhibited an OR of 2.06 (*p* = 0.001). Additionally, the Prothrombin (PT) G20210A homozygous mutation was identified as a risk factor, with an OR of 1.92 (*p* = 0.003). Lastly, the PAI-1 4G/4G homozygous variant also posed a significant risk, with an OR of 1.36, supported by a *p*-value of 0.001, as presented in Table 6 and Figure 1.

The MTHFR A1298C homozygous mutation emerged as a significant risk factor, presenting an OR of 3.79 with a coefficient of 1.01 (*p*-value < 0.001). Similarly, the β-Fibrinogen 455 G>A heterozygous mutation was identified as a substantial risk factor for late pregnancy loss, with an OR of 2.20 (*p*-value = 0.002). Furthermore, the MTHFR A1298C heterozygous mutation also significantly increased the risk of late pregnancy loss, showcasing an OR of 2.65, with a *p*-value lower than 0.001.

The Factor XIII G1002T heterozygous mutation, while showing a lower OR of 1.18 and a coefficient of 0.69, was still significantly associated with late pregnancy loss (*p*-value = 0.023). Additionally, the Plasminogen Activator Inhibitor-1 (PAI-1) 4G/5G homozygous mutation was significantly correlated with late pregnancy loss, exhibiting an OR of 2.85, a coefficient of 1.05, and a 95% CI of 1.70 to 4.72, with a *p*-value of less than 0.001.

**Figure 2:**
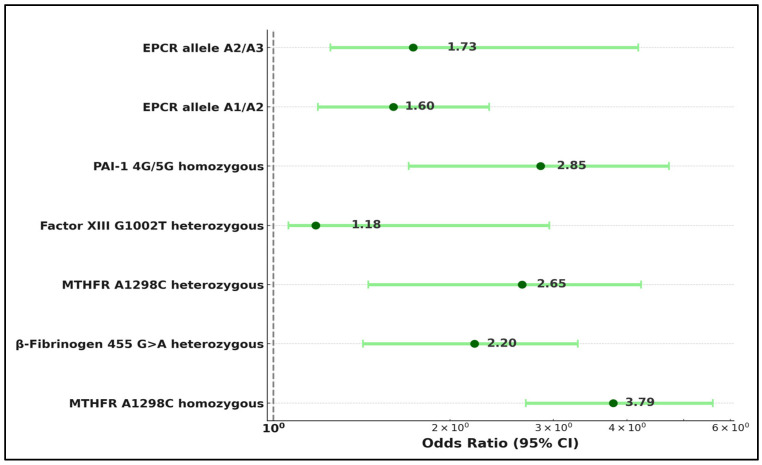
Risk factor analysis for late pregnancy loss.

Lastly, the Endothelial Protein C Receptor (EPCR) alleles A1/A2 and A2/A3 were also identified as significant risk factors, with ORs of 1.60 and 1.73, coefficients of 0.47 and 0.66, and 95% CIs of 1.19 to 2.33 and 1.25 to 4.18, respectively, supported by *p*-values of 0.012 and 0.010, as described in Table 7 and Figure 2.

First and foremost, the Factor V Leiden G1691A homozygous mutation stood out as a highly significant risk factor for multiple miscarriage, with an OR of 3.04 (*p*-value < 0.001). Similarly, the Prothrombin G20210A homozygous mutation emerged as a substantial risk factor with an OR of 3.17 (*p*-value < 0.001). The MTHFR C677T heterozygous mutation was also identified as a significant risk factor for multiple miscarriage, presenting an OR of 2.52 (*p*-value < 0.001).

**Figure 3:**
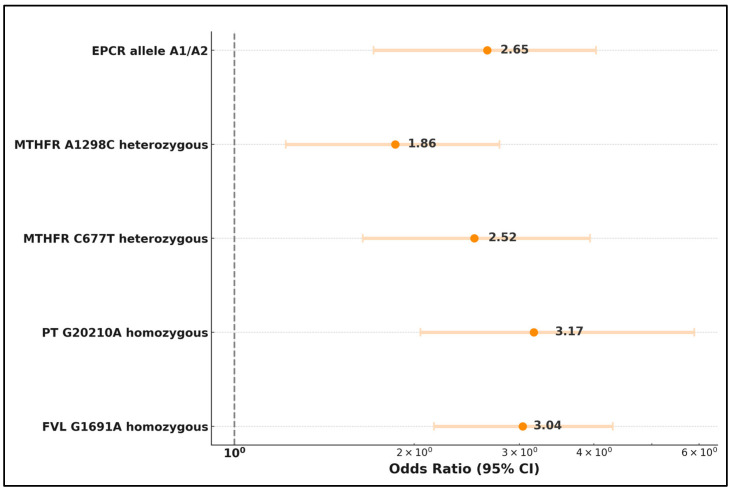
Risk factor analysis for multiple miscarriage.

Furthermore, the MTHFR A1298C heterozygous mutation exhibited a notable association with multiple miscarriages, with an OR of 1.86 (*p*-value = 0.002). Lastly, the Endothelial Protein C Receptor allele A1/A2 was identified as a robust risk factor for multiple miscarriages, presenting an OR of 2.65 (*p*-value < 0.001), emphasizing its substantial role in increasing the likelihood of experiencing multiple miscarriages, as shown in Table 8 and Figure 3.

## 4. Discussion

The study results reveal significant genetic factors associated with pregnancy loss, providing insights into both early and late losses, as well as multiple miscarriages. Notably, the MTHFR C677T homozygous mutation was significantly linked to early pregnancy loss, while the PAI-1 4G/5G homozygous mutation was associated with a higher risk of early loss. The interaction between the homozygous mutations of Factor V Leiden (FVL G1691A) and MTHFR C677T suggests a synergistic effect contributing to multiple miscarriages, emphasizing the need to consider combined genetic influences.

The distribution of early and late miscarriage cases in this study underscores the influence of targeted recruitment and screening for genetic thrombophilia, which may not reflect the broader epidemiological trends of miscarriage. This targeted approach allowed for a concentrated examination of thrombophilia’s impact on pregnancy loss, highlighting the need for further research into tailored screening strategies in populations at high risk for thrombotic disorders. Late pregnancy loss exhibited its distinct risk factors, with the MTHFR A1298C homozygous mutation and β-Fibrinogen 455 G>A heterozygous mutation standing out as significant contributors. The study also highlighted the relevance of genetic interactions, such as the PT G20210A homozygous mutation and β-Fibrinogen 455 G>A heterozygous mutation, in late pregnancy loss.

In the context of multiple miscarriages, the Factor V Leiden G1691A homozygous mutation, Prothrombin G20210A homozygous mutation, MTHFR C677T heterozygous mutation, MTHFR A1298C heterozygous mutation, and EPCR allele A1/A2 were identified as substantial risk factors. These findings underscore the intricate interplay of genetics in determining the risk of multiple miscarriages. Further research in this area can enhance our understanding of pregnancy loss and inform strategies for its prevention and management.

In alignment with our study, the research conducted by Iordache et al. in Romania underscores the profound influence of thrombophilia mutations on the gestational timing of recurrent pregnancy loss [12]. Their findings demonstrated that Factor V Leiden (FVL) homozygosity and antiphospholipid syndrome (APS) antibodies were significantly more prevalent in first-trimester pregnancy losses, illustrating a heightened thrombotic risk early in gestation. Notably, Iordache et al. quantified the risk, revealing that the strongest independent risk factors for first-trimester losses were FVL and prothrombin (PT) compound mutations, with a 3-times-higher risk of miscarriage, followed by FVL homozygous mutation, with an OR of 3.66, and APS antibodies, which increased the risk of miscarriage by 4 times. For second-trimester losses, the most significant risks were associated with FVL and PT compound mutations (OR = 3.24), Glycoprotein Ia polymorphism (OR = 3.61), and APS antibodies (OR = 3.85). Similarly to our study, these objective findings offer critical insights into the thrombophilia risk factors influencing early and late pregnancy loss, resonating with the patterns observed in our analysis and highlighting the nuanced role of specific genetic markers in RPL.

The prevalence and impact of thrombophilia on recurrent pregnancy loss exhibit significant variability across studies, with Nahas et al. [42] reporting a significant prevalence of FVL (20.9%) and protein S deficiency (19.0%) among women with recurrent miscarriages, highlighting that Enoxaparin treatment increased live birth rates, especially for those with four or more miscarriages. This suggests that a targeted approach to antithrombotic therapy could be beneficial, particularly in high-risk groups. In contrast, Yousif et al. [14] focused on first-trimester miscarriages, noting a higher incidence of pregnancy complications in this period, attributed to thrombophilia, without providing specific prevalence rates for thrombophilia disorders.

Parand et al. [43] contrasted these findings by not observing significant differences in the frequency of common thrombophilia mutations (FVL and PT G20210A) between RPL patients and controls, though they reported a higher frequency of protein S deficiency in the RPL group. Hansda et al. [44] further built on the association between thrombophilia and pregnancy loss, noting a 64.1% prevalence of thrombophilia defects in their study group, with protein S deficiency being the most common (50.9%). They also identified a strong association of pregnancy complications with protein S deficiency (87.5%) and elevated factor VIII levels (66.6%). These studies collectively underscore the complexity of thrombophilia’s impact on RPL, highlighting the importance of comprehensive screening and personalized treatment plans, especially for those with identified thrombophilia disorders.

Other authors highlight the prevalence of thrombophilia gene polymorphisms in women with RPL, reporting significant associations with factor V Leiden and prothrombin G20210A mutations. Specifically, Barut et al. [45] found heterozygous factor V Leiden H1299R and prothrombin G20210A mutations were significantly associated with RPL in Turkish women, emphasizing the utility of diagnosing these polymorphisms. Skrzypczak et al. [46] identified a 9.1% prevalence of inherited thrombophilia among Polish women with pregnancy loss, with factor V Leiden mutations being most prevalent (73%) in cases of intrauterine fetal demise.

Contrastingly, Nassour-Mokhtari et al. [47] observed an 8.33% prevalence of factor V Leiden in Algerian women with RPL, notably associated with second-trimester losses, while prothrombin G20210A mutation did not show a significant correlation with RPL. Ali et al. [48] reported a higher prevalence of hereditary thrombophilia in Pakistani women with more than three pregnancy losses (4%), demonstrating a strong association between hereditary thrombophilia and increased numbers of pregnancy losses. These findings collectively suggest that factor V Leiden and prothrombin G20210A mutations play a significant role in RPL across different populations, with specific thrombophilia profiles potentially influencing the timing and recurrence of pregnancy losses, highlighting the need for tailored screening and management approaches.

Liu et al.’s [49] systematic review and meta-analysis revealed that specific hereditary thrombophilia, notably the FVL mutation, PT gene mutation, and protein S deficiency, significantly elevates the risk of recurrent pregnancy loss (RPL). With an odds ratios of 2.44 for FVL, 2.08 for PT, and a notably high 3.45 for protein S deficiency, their findings underscore the substantial impact of these genetic factors on RPL. This comprehensive analysis, involving over 30,000 individuals across 89 studies, highlights the critical need for thrombophilia screening in women experiencing RPL, especially those with these specific genetic predispositions.

Echoing Liu et al., Kovalevsky et al. [50] further confirmed the association between hereditary thrombophilia and RPL, with their meta-analysis showing that carriers of FVL or G20210A mutations have double the risk (OR = 2.0) of suffering two or more miscarriages compared to non-carriers. This consistent identification of FVL and PT gene mutation as significant risk factors for RPL across both studies emphasizes the role of genetic screening in the management of RPL. By quantifying the risk associated with hereditary thrombophilia, this meta-analysis reinforces the recommendation for testing these mutations in women with RPL, supporting a targeted approach to reducing miscarriage risk.

The ALIFE2 trial [51] marks a pivotal moment in the ongoing debate over the effectiveness of low-molecular-weight heparin in treating recurrent pregnancy loss among women with hereditary thrombophilia. Despite LMWH’s routine prescription, based on the premise that its anticoagulant properties potentially mitigate thrombophilia-related pregnancy complications, the ESHRE’s 2022 guideline updates introduced a conditional recommendation against its use, citing low certainty in evidence [52]. This backdrop set the stage for the ALIFE2 trial, aiming to address the critical knowledge gap regarding LMWH’s efficacy in this context.

The trial spanned over 8.5 years and involved 326 women, representing a significant endeavor to empirically evaluate LMWH’s role in managing recurrent pregnancy loss tied to inherited thrombophilia. The dedication to this extensive research effort underscores the complexity of making informed treatment decisions in the face of ambiguous evidence. Strandell and Hellgren’s highlights of the ALIFE2 trial not only question the continued prescription of LMWH but also suggest a broader reconsideration of treatment protocols for women with hereditary thrombophilia experiencing recurrent pregnancy loss, pushing towards evidence-based practices in a field marked by historical uncertainty [53].

Other aspects that should be considered when discussing genetic testing for thrombophilia are the geographic and population characteristics in the study cohort, which might differ widely when compared to other studies. Therefore, some significant insights into the prevalence of inherited thrombophilia among Romanian women were described recently in two studies. The study by Iordache et al. [12] included a comprehensive analysis of 211 pregnancies, where 157 experienced first-trimester miscarriage and 54 second-trimester pregnancy loss, revealing that FVL homozygosity and APS antibodies were significantly more prevalent in women experiencing first-trimester pregnancy losses, while Protein C deficiency and Glycoprotein Ia polymorphism were more common in the second-trimester losses. On the other hand, Nitu et al. [54] provided a cross-sectional analysis of 238 women with thrombophilia and a history of RPL, distinguishing between those who did not give birth and those who achieved pregnancy and gave birth. Their findings highlight significant differences in emotional and relational outcomes, with the former group experiencing greater intimacy problems, higher stress levels, and lower marital satisfaction. Even though the exact prevalence is not mentioned, it is known that the population served by these clinics is about 300 thousand inhabitants, and approximately 70,000 women of reproductive age.

While current guidelines do not universally recommend screening for genetic thrombophilia following pregnancy loss, our findings suggest the potential value of a selective approach for patients with recurrent losses or those with familial histories of thrombotic events [17]. This tailored strategy may uncover actionable thrombophilia disorders, enabling personalized interventions that could mitigate the risk of future pregnancy losses, such as a personalized approach to antithrombotic prophylaxis in pregnancy. Our data underscore the importance of careful evaluation and management of pregnant patients who may potentially benefit from prophylactic anticoagulation, reinforcing the need for guidelines to be adaptable to emerging evidence on the efficacy of such interventions.

One of the most important limitations of this retrospective cohort study is the potential for selection bias. The study was conducted at a single academic medical center, which may not fully represent the broader population of pregnant women who experience miscarriages. Participants were selected based on their availability in the hospital’s database, and there may be a bias towards including women who sought medical attention for miscarriages, potentially excluding those who did not seek medical care or were managed elsewhere. This selection bias could affect the generalizability of the study’s findings to the wider population of pregnant women who experience miscarriages. Another limitation to consider is the retrospective nature of the study. It is important to note that the retrospective nature of this study did not allow for the exclusion of patients based on their medication use, including those on Enoxaparin and aspirin for comorbid conditions.

Additionally, the study’s reliance on historical data may limit the ability to establish causal relationships between genetic thrombophilia and pregnancy loss as it cannot account for temporal factors or changes in medical practice over time. The study was unable to include a control group of pregnant women with normal ongoing pregnancies due to the financial barriers associated with genetic testing in Romania, where such tests are not covered by government or insurance programs, limiting our ability to comprehensively assess the prevalence of MTHFR mutations and other thrombophilia mutations among the wider population. To address this limitation, it is important to take into consideration the prevalence of these mutations in healthy populations, particularly among women of Caucasian origin with healthy pregnancies, to provide context for our findings despite the absence of a direct control group. Among Caucasians, FVL stands as the most common inherited thrombophilia, but it is infrequently found in non-Caucasians. It can be passed down as a heterozygous mutation, affecting around 4.7% of Europeans or individuals of European descent, or as a homozygous mutation, found in approximately 0.06–0.25% of the population. Its prevalence rises to 19% among individuals with DVT and 28.4% in cases of recurrent VTE [55].

Another limitation of this study is the absence of fetal genetic testing, such as karyotyping and Chromosomal Microarray Analysis, in our records, which might have excluded certain causes of miscarriage. Therefore, considering the most common cause of miscarriage in the first trimester is chromosomal abnormalities, the findings of thrombophilia mutations could be just coincidental in this case [56]. Furthermore, small sample sizes can limit the statistical power to detect significant associations and increase the risk of Type II errors. Additionally, while the study controlled for several potential confounding factors, there may still be unmeasured or residual confounders that were not considered in the analysis.

## 5. Conclusions

In conclusion, our study provides essential insights into the role of thrombophilia mutations in pregnancy losses among Romanian women, shedding light on the intricate interactions between specific genetic variants and their association with different types of miscarriages. Notably, the synergistic effects of certain mutations, such as Factor V Leiden and MTHFR, underscore the importance of considering multiple genetic factors in miscarriage risk assessment. Additionally, our findings identify significant risk factors for early and late pregnancy loss, offering valuable information for the development of evidence-based guidelines for miscarriage management tailored to specific genetic profiles. These discoveries have the potential to reduce the burden of miscarriage on affected women and their families by enabling more personalized and targeted approaches to care.

## Figures and Tables

**Table 1:** Patients’ demographics.

Variables	Early Pregnancy Loss (*n* = 103)	Late Pregnancy Loss (*n* = 52)	*p*-Value
BMI (mean ± SD)	22.8 ± 4.5	23.6 ± 4.4	0.294
Age group			0.081
20–30 years	39 (35.14%)	24 (54.55%)	
31–40 years	51 (45.95%)	27 (61.36%)	
>40 years	13 (11.71%)	1 (2.27%)	
Place of residence			0.092
Urban	82 (79.6%)	35 (67.3%)	
Rural	21 (20.4%)	17 (32.7%)	
Education			0.163
Elementary school	46 (44.7%)	25 (48.1%)	
High school	1 (1.0%)	3 (5.8%)	
College/university	56 (54.4%)	24 (46.2%)	
Number of miscarriages			0.136
1	39 (37.9%)	20 (38.5%)	
2	45 (43.7%)	18 (34.6%)	
3	15 (14.6%)	14 (26.9%)	
≥4	4 (3.9%)	0 (0.0%)	
Children			0.837
Yes	11 (10.7%)	5 (9.6%)	
No	92 (89.3%)	47 (90.4%)	

SD—standard deviation.

**Table 2:** Patients’ medical history.

Variables	Early Pregnancy Loss (*n* = 103)	Late Pregnancy Loss (*n* = 52)	*p*-Value
Pregnancy			0.354
Naturally conceived pregnancy	82 (79.6%)	39 (75.0%)	
Pregnancy achieved through IVF	17 (16.5%)	8 (15.4%)	
Pregnancy achieved through artificial insemination	3.9 (3.9%)	5 (9.6%)	
Contraceptive use before pregnancy	62 (60.2%)	32 (61.5%)	0.871
Comorbid conditions			
Personal history of thrombosis	27 (26.2%)	12 (23.1%)	0.670
Family history of thrombosis	40 (38.8%)	23 (44.2%)	0.518
Thyroid disease	14 (13.6%)	9 (17.3%)	0.539
Hypertension	12 (11.7%)	2 (3.8%)	0.109
Diabetes	16 (15.5%)	2 (3.8%)	0.031
Medication during pregnancy			
Folic Acid	103 (100%)	52 (100%)	–
Aspirin	83 (80.6%)	46 (88.5%)	0.215
Enoxaparin	103 (100%)	52 (100%)	–
Laboratory analysis			
Anti-factor X (IU/mL) first trimester	0.33 ± 0.11	0.29 ± 0.07	0.018
Anti-factor X (IU/mL) second trimester	0.32 ± 0.07	0.31 ± 0.07	0.402
Anti-factor X (IU/mL) third trimester	0.36 ± 0.06	0.37 ± 0.09	0.411
D-dimers (ng/mL) first trimester	0.36 ± 0.25	0.35 ± 0.34	0.853
D-dimers (ng/mL) second trimester	1.59 ± 0.60	1.58 ± 0.42	0.914
D-dimers (ng/mL) third trimester	2.40 ± 0.86	2.25 ± 0.57	0.257

IVF—in vitro fertilization; IU—International Units.

**Table 3:** Thrombophilia genetic assessment by trimester of pregnancy loss.

Variables	Early Pregnancy Loss (*n* = 103)	Late Pregnancy Loss (*n* = 52)	*p*-Value
FVL G1691A homozygous	12 (11.7%)	2 (3.9%)	0.109
FVL G1691A heterozygous	38 (36.9%)	6 (11.5%)	<0.001
FVL A4070G-HR2 homozygous	0 (0.0%)	0 (0.0%)	–
FVL A4070G-HR2 heterozygous	9 (8.7%)	1 (1.9%)	0.102
PT G20210A homozygous	27 (26.2%)	1 (1.9%)	<0.001
PT G20210A heterozygous	14 (13.6%)	4 (7.7%)	0.279
MTHFR C677T homozygous	13 (12.6%)	0 (0.0%)	0.007
MTHFR C677T heterozygous	67 (65.1%)	15 (28.8%)	<0.001
MTHFR A1298C homozygous	13 (12.6%)	5 (9.6%)	0.581
MTHFR A1298C heterozygous	62 (60.2%)	9 (17.3%)	<0.001
PAI-1 4G/5G homozygous	31 (30.1%)	6 (11.5%)	0.010
PAI-1 4G/5G heterozygous	49 (47.6%)	11 (21.2%)	0.001
PAI-1 4G/4G homozygous	15 (14.6%)	1 (1.9%)	0.014
PAI-1 4G/4G heterozygous	5 (4.9%)	0 (0.0%)	0.106
Glycoprotein Iib/IIIa T1565C homozygous	15 (14.6%)	8 (15.4%)	0.891
Glycoprotein Iib/IIIa T1565C heterozygous	11 (10.7%)	4 (7.67%)	0.552
Factor XIII G1002T homozygous	11 (10.7%)	9 (17.3%)	0.245
Factor XIII G1002T heterozygous	32 (31.1%)	20 (38.5%)	0.357
β-Fibrinogen 455 G>A homozygous	12 (11.7%)	4 (7.7%)	0.444
β-Fibrinogen 455 G>A heterozygous	17 (16.5%)	16 (30.8%)	0.040
EPCR allele A1/A2	43 (49.5%)	19 (21.2%)	0.531
EPCR allele A2/A3	6 (5.8%)	6 (11.5%)	0.209
EPCR allele A2/A2	11 (10.7%)	2 (3.9%)	0.147

FVL—Factor V Leiden; PT—Prothrombin; MTHFR—Methylenetetrahydrofolate Reductase; PAI-1—Plasminogen Activator Inhibitor-1; EPCR—Endothelial Protein C Receptor.

**Table 4:** Thrombophilia genetic assessment by number of miscarriages.

Variables	Single Miscarriage (*n* = 59)	Multiple Miscarriages (*n* = 96)	*p*-Value
FVL G1691A homozygous	1 (1.7%)	13 (13.5%)	0.012
FVL G1691A heterozygous	16 (27.1%)	28 (29.2%)	0.783
FVL A4070G-HR2 homozygous	0 (0.0%)	0 (0.0%)	–
FVL A4070G-HR2 heterozygous	3 (5.1%)	7 (7.3%)	0.587
PT G20210A homozygous	12 (20.3%)	16 (16.7%)	0.016
PT G20210A heterozygous	4 (13.6%)	14 (11.5%)	0.140
MTHFR C677T homozygous	5 (8.5%)	8 (8.3%)	0.975
MTHFR C677T heterozygous	24 (40.7%)	58 (60.4%)	0.017
MTHFR A1298C homozygous	6 (10.2%)	12 (12.5%)	0.660
MTHFR A1298C heterozygous	21 (35.6%)	50 (52.1%)	0.045
PAI-1 4G/5G homozygous	14 (23.7%)	23 (24.0%)	0.974
PAI-1 4G/5G heterozygous	20 (33.9%)	40 (41.7%)	0.334
PAI-1 4G/4G homozygous	6 (10.2%)	10 (10.4%)	0.960
PAI-1 4G/4G heterozygous	2 (3.4%)	3 (3.1%)	0.927
Glycoprotein Iib/IIIa T1565C homozygous	8 (13.6%)	15 (15.6%)	0.725
Glycoprotein Iib/IIIa T1565C heterozygous	7 (11.9%)	8 (8.3%)	0.470
Factor XIII G1002T homozygous	10 (17.0%)	10 (10.4%)	0.238
Factor XIII G1002T heterozygous	21 (35.6%)	31 (32.3%)	0.672
β-Fibrinogen 455 G>A homozygous	6 (10.2%)	10 (10.4%)	0.960
β-Fibrinogen 455 G>A heterozygous	13 (22.0%)	20 (20.8%)	0.859
EPCR allele A1/A2	15 (25.4%)	47 (49.0%)	0.003
EPCR allele A2/A3	4 (6.8%)	8 (8.3%)	0.725
EPCR allele A2/A2	5 (8.5%)	8 (8.3%)	0.975

FVL—Factor V Leiden; PT—Prothrombin; MTHFR—Methylenetetrahydrofolate Reductase; PAI-1—Plasminogen Activator Inhibitor-1; EPCR—Endothelial Protein C Receptor.

**Table 5:** Interactions between genetic markers for multiple miscarriage.

Factor 1	Factor 2	Coefficient	*p*-Value
FVL G1691A homozygous	MTHFR C677T homozygous	3.42	0.003
PT G20210A homozygous	β-Fibrinogen 455 G>A heterozygous	1.98	0.009
MTHFR A1298C heterozygous	PAI-1 4G/5G heterozygous	1.65	0.015
FVL G1691A heterozygous	EPCR allele A1/A2	2.10	0.006
MTHFR C677T homozygous	Glycoprotein Iib/IIIa T1565C heterozygous	1.77	0.021

FVL—Factor V Leiden; PT—Prothrombin; MTHFR—Methylenetetrahydrofolate Reductase; PAI-1—Plasminogen Activator Inhibitor-1; EPCR—Endothelial Protein C Receptor.

**Table 6:** Risk factor analysis for early pregnancy loss.

Risk Factors	Odds Ratio	Coefficient	95% CI	*p*-Value
PAI-1 4G/5G homozygous	3.01	0.70	[1.39, 5.34]	<0.001
FVL G1691A heterozygous	1.85	0.62	[1.22, 2.48]	0.002
MTHFR A1298C heterozygous	1.55	0.44	[1.05, 2.26]	0.028
MTHFR C677T homozygous	2.38	0.56	[1.69, 4.72]	<0.001
MTHFR C677T heterozygous	2.06	0.69	[1.50, 2.65]	0.001
PT G20210A homozygous	1.92	0.64	[1.35, 2.68]	0.003
PAI-1 4G/4G homozygous	1.36	0.73	[1.11, 4.37]	0.001

FVL—Factor V Leiden; PT—Prothrombin; MTHFR—Methylenetetrahydrofolate Reductase; PAI-1—Plasminogen Activator Inhibitor-1; CI—Confidence Interval.

**Table 7:** Risk factor analysis for late pregnancy loss.

Risk Factors	Odds Ratio	Coefficient	95% CI	*p*-Value
MTHFR A1298C homozygous	3.79	1.01	[2.69, 5.60]	<0.001
β-Fibrinogen 455 G>A heterozygous	2.20	0.79	[1.42, 3.30]	0.002
MTHFR A1298C heterozygous	2.65	0.83	[1.45, 4.23]	<0.001
Factor XIII G1002T heterozygous	1.18	0.69	[1.06, 2.95]	0.023
PAI-1 4G/5G homozygous	2.85	1.05	[1.70, 4.72]	<0.001
EPCR allele A1/A2	1.60	0.47	[1.19, 2.33]	0.012
EPCR allele A2/A3	1.73	0.66	[1.25, 4.18]	0.010

MTHFR—Methylenetetrahydrofolate Reductase; PAI-1—Plasminogen Activator Inhibitor-1; EPCR—Endothelial Protein C Receptor.

**Table 8:** Risk factor analysis for multiple miscarriage.

Risk Factors	Odds Ratio	Coefficient	95% CI	*p*-Value
FVL G1691A homozygous	3.04	0.88	[2.16, 4.30]	<0.001
PT G20210A homozygous	3.17	1.13	[2.05, 5.89]	<0.001
MTHFR C677T heterozygous	2.52	0.92	[1.64, 3.94]	0.001
MTHFR A1298C heterozygous	1.86	0.62	[1.22, 2.78]	0.002
EPCR allele A1/A2	2.65	0.98	[1.71, 4.03]	<0.001

FVL—Factor V Leiden; PT—Prothrombin; MTHFR—Methylenetetrahydrofolate Reductase; EPCR—Endothelial Protein C Receptor.

## Data Availability

Data available on request.
